# Disentangling homeologous contigs in allo-tetraploid assembly: application to durum wheat

**DOI:** 10.1186/1471-2105-14-S15-S15

**Published:** 2013-10-15

**Authors:** Vincent Ranwez, Yan Holtz, Gautier Sarah, Morgane Ardisson, Sylvain Santoni, Sylvain Glémin, Muriel Tavaud-Pirra, Jacques David

**Affiliations:** 1 Montpellier SupAgro, UMR AGAP, F-34060 Montpellier, France; 2 INRA, UMR AGAP, F-34060 Montpellier, France; 3 Institut des Sciences de l'Evolution de Montpellier (ISE-M), UMR 5554 CNRS Université Montpellier II, place E. Bataillon, CC 064, 34 095 Montpellier cedex 05, France

## Abstract

**Background:**

Using Next Generation Sequencing, SNP discovery is relatively easy on diploid species and still hampered in polyploid species by the confusion due to homeology. We develop HomeoSplitter; a fast and effective solution to split original contigs obtained by RNAseq into two homeologous sequences. It uses the differential expression of the two homeologous genes in the RNA. We verify that the new sequences are closer to the diploid progenitors of the allopolyploid species than the original contig. By remapping original reads on these new sequences, we also verify that the number of valuable detected SNPs has significantly increased.

Thirty accessions of the tetraploid *durum *wheat (*Triticum turgidum*, A and B genomes) were sequenced in pooled cDNA libraries. Reads were assembled in a *de novo *durum assembly. Transcriptomes of the diploid species, *Aegilops speltoides *(close B genome) and *Triticum urartu (*A genome) were used as reference to benchmark the method.

**Results:**

HomeoSplitter is a fast and effective solution to disentangle homeologous sequences based on a maximum likelihood optimization. On a benchmark set of 2,505 clusters containing homologous sequences of *urartu, speltoides *and durum, HomeoSplitter was efficient to build sequences closer to the diploid references and increased the number of valuable SNPs from 188 out of 1,360 SNPs detected when mapping the reads on the *de novo *durum assembly to 762 out of 1,620 SNPs when mapping on HomeoSplitter contigs.

**Conclusions:**

The HomeoSplitter program is freely available at http://bioweb.supagro.inra.fr/homeoSplitter/. This work provides a practical solution to the complex problem of disentangling homeologous transcripts in allo-tetraploids, which further allows an improved SNP detection.

## Introduction

Unravelling genome diversity allows addressing basic evolutionary questions as well as giving tools for applied sciences such as plant breeding and livestock management. Single nucleotide polymorphism (SNP) is now a common avenue since Next Generation Sequencing (NGS) technology provides a rapid, cheap and direct access to the genome. In a close future, diversity surveys, high-density mapping, genome wide association studies or genomic selection will be democratized for non model species or orphan crops [1].

SNP discovery relies on mapping re-sequencing data of a diversity panel on a reference sequence. As complete genome sequences will still require a lot of efforts and international consortia, *e.g*., the International Wheat Genome Sequencing Consortium (IWGSC, http://www.wheatgenome.org), sequencing a reduced but repeatable portion of the genome appears as a provisory amenable approach. Reduction of genome complexity can be achieved *via *sequencing cDNA obtained in standardised conditions (RNAseq) [2,3]. Difficulties for the use of RNAseq for SNP discovery come from alternative splicing, differential expression between genes leading to poor coverage of lowly expressed genes, weak evolutionary signal for detecting paralogous genes and transcription errors [4]. Nevertheless, this approach has though proven its efficiency to genotype few individuals on several thousands of genes for non model organisms [5] and to produce SNP data base in many species [2,3].

Plants in contrast to animals are often of recent polyploid origin [6,7]. In allopolyploids, two or more sub-genomes are present and SNP discovery and genotyping using cDNA is complicated by the parallel expression of homeologous copies of the same genes. Observed sequence variation may be due to divergence between homeologous copies or to intra-genome (homologous) allelic polymorphism. Reads of cDNA homeologous copies are thus frequently assembled in the same reference gene (either from *de novo *reference assembly or on the whole genome sequence) and genotypes commonly show excess of spurious heterozygous sites [3]. Genomic sequences are submitted to the same confusion effect [8]. The SNPs with excess of heterozygosity are usually simply discarded [8], lowering down the yield in workable SNPs. This specific situation of allopolyploidy is superimposed to other sources of errors also existing in diploid species such as undetected paralogy or copy number variation [1]. Developing a good method for tackling this issue is crucial for important allo-polyploid crops (potato, durum and bread wheat, cotton, canola, tobacco and peanuts [1]).

Computational methods for coping with paralogy induced by allopolyploidy are dealing first with assembly stringency. If the divergence between the two sub-genomes is tight, a lot of confusion will still be present in assembly. Increasing stringency also leads to a reduced length of *de novo *assembled contigs and to the possible, undesirable, separation of allelic variants. The use of the diploid progenitors may be of great help for the identification of sub-genomes specificity [9]. When this resource is not available (when at least one of them is not available or identified) or when the diploid assembly does not cover the whole transcriptome of the allo-polyploid target species, there is no real satisfying method. Identifying homeologous *de novo *contigs and splitting them into two new sequences could help resolving the issue of heterozygous excess due to homeologous confusion. Remapping reads on these two new sequences may provide a more clearcut discrimination of valuable homologous SNPs.

### State of the art

The problem of reconstructing the two homeologous copies merged in a single contig can be seen as a variant of the phasing problem that aims at reconstructing the different haplotypes merged in a single contig. This latter problem has been extensively studied since the seminal work of Clark in 1990 [10]. Existing algorithmic solutions can be grouped in two main categories: i) the genotyping based approaches that infer haplotypes based on the genotyping of several accessions and ii) the co-occurring based approaches that infer haplotypes based on nucleotide co-occurrence on the same sequenced fragment. Those two strategies will be briefly presented here with respect to their potential use to separate homeologous copies (see [11] for a review about their relative efficiencies).

Genotyping based approaches mostly rely on population genetics theory to identify a set of haplotypes in a parsimonious [12-14]; maximum likelihood [15] or bayesian framework [16,17]. Such approaches are hardly adaptable to disentangle homeologous copies since in this latter case i) the mix of homeologous copies will bias the genotyping inference on which they rely ii) their underlying model assume coalescences of haplotypes within a single locus whereas homeologous copies result from a single duplication event. For instance, in the simplest Haplotype reconstruction described by Clark in 1990 [10]; the algorithm starts building reliable haplotypes by identifying individuals with no (or only few) heterozygous sites whereas it is precisely the reverse situation (heterozygous excess) that suggests homeology.

Phasing method based on nucleotide co-occurrence in the same reads (or ESTs) mostly ignore the underlying biological model and rely on combinatorial and graph theory to tackle the problem [18-20]. The fact that two nucleotides appear on the same reads is a strong indication that they belong to a same haplotype. This indication can however be erroneous on low coverage regions due to sequencing errors. Moreover, the fact that reads come from the same accession is not taken into account, so that even if an accession is homozygous CC and TT at two distant sites, phasing is ignored unless some reads overlap the two sites for this accession. In addition, SNP density must be sufficiently high to ensure that many reads contain two or more SNPs all along the contig [21]. Such methods are thus adapted for high coverage sequencing with long reads and/or high SNP density [11]. For all those reasons, they are often used as post-processing of genotyping based-phasing as for instance in Haplotype Improver [18]. A recent work uses a different approach that simultaneously assembles reads and predicts haplotypes using colored De Bruijn graphs [22], a dedicated variant of the De Bruijn graphs [23]. The associated CORTEX software [24] uses individual information to predict haplotypes. Disentangling homeologous copies can be seen as a quite similar problem except that, in allo-polyploid species, no easy solution exists to allocate a distinct colour to reads coming from the two homeologous copies with the exception of chromosome sorting [25].

In this paper, we propose a solution, dedicated to allo-tetraploid species, that uses the nucleotide counts observed at each site, which is much more informative than using just accession genotypes but much more lightweight than using full read information. This allows us to design a fast dedicated solution that identifies contigs for which a heterozygous excess may sign the assembly of two homeologous/paralogous copies. On these contigs, we propose a likelihood model-based method to rebuild mixed contigs in two new sequences based on their differential expression, a largely documented phenomenon on homeologous copies [26-28]. We test the new sequences for their distances to the diploid progenitors of the allo-tetraploid species and verify their efficiency to map properly reads and to provide a significant increased amount of new valuable SNPs compared to *de novo *mapping method.

The method is implemented in the HomeoSplitter software, tested and evaluated on durum wheat (*Triticum turgidum *L.), an allo-tetraploid inbreeding species and is validated on the reference transcriptomes of its two diploid progenitors.

## Material

### Durum wheat genomes

Allo-tetraploid wheats (*T. turgidum *L.) originate from the spontaneous hybridization of two ancestral diploid species (2n = 4X = 28, A and B genomes). The current descent of its A progenitor has been identified as the diploid *T. urartu *[29] which genome is still lowly differentiated from the A genome of *durum. Aegilops speltoides *is the most closely related extant species to the B genome of tetraploid wheat [30], the real ancestor of the B genome being either extinct or not yet discovered. The divergence of the *Triticum/Aegilops *alliance is approximately dated between 2.5 and 4.5 million years ago [31]. The origin of AB tetraploid wheat was reported to have originated ca. 0.36 million years ago [32].

### Accessions, cDNA extraction and preparation

The diploid reference transcriptomes were assembled from the cDNA libraries obtained on a *Triticum urartu *accession (*dv1792*, kindly provided by Pr J. Dvorak, UC Davis) and an *Ae. speltoides *var. *speltoides *accession from Turkey (USDA PI 542268, http://www.ars-grin.gov/npgs/). These two lines will be further called *urartu *and *speltoides *accessions.

The 30 durum accessions analysed were issued from single seed descents of plants sampled in a base broadening population of durum wheat (see additional file 1). The averaged observed heterozygosity of the 30 descents was 0.022 on 30 microsatellite loci. Taking allelic frequencies into account, the fixation index, *Fis *[33] was estimated at 0.95 on the whole sample (data not shown). These 30 accessions will be called durum wheat accessions.

For each accession, we obtained sequence data by RNAseq procedure, mainly consisting in mRNA extraction and purification, libraries construction, mixing, and sequencing using the Illumina mRNA-Seq, paired-end indexed protocol on a HiSeq2000 sequencer. The protocol is detailed in additional file 2.

### Reads assembly and mapping

**Figure 1:**
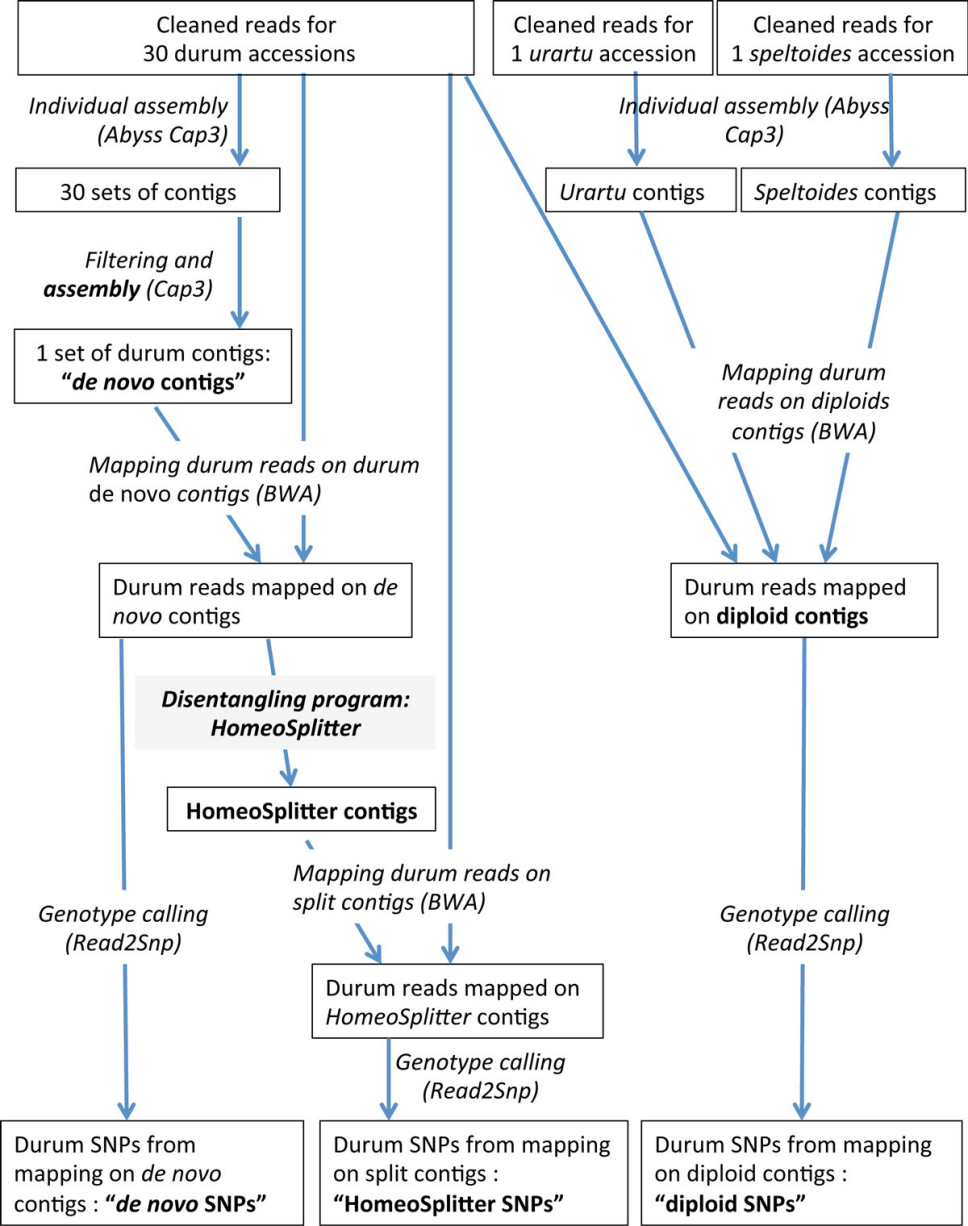
**Overview of the SNPs identification pipeline** .

The whole pipeline described in this section is schematically summarized in Figure 1 and detailed in the additional file 3. *Urartu *and *speltoides *accessions were assembled in respectively *urartu *and *speltoides *contigs, and durum accessions were assembled in *de novo *contigs. Durum reads were thus mapped on three different references: 1) on *urartu *and *speltoides de novo *contigs giving "diploids SNPs", 2) on durum *de novo *contigs, giving "*de novo *SNPs", 3) on the newly recomposed contigs ("HomeoSplitter contigs") using HomeoSplitter (see below) giving "HomeoSplitter SNPs".

### Benchmark constitution

To evaluate and validate our HomeoSplitter approach, we clustered *de novo *and *diploid *contigs using CAP3 [34]. Though CAP3 is not initially designed for sequence clustering it works particularly well in our case since sequences to be clustered are highly similar, it also has the advantage of directly providing us with an alignment of those sequences. We then kept only clusters containing simultaneously one contigs of *urartu*, one of *speltoides *and one or two of *durum having at least *100 nucleotides of overlap with the diploid contigs.

## Methods

HomeoSplitter aims at identifying contigs that result from a mixing of reads coming from homeologs and replacing each of those contigs by two new contigs (one per homeolog). First, HomeoSplitter identifies problematic sites and contigs based on their excess of observed heterozygosity. Indeed, at sites where the apparent polymorphism is actually due to divergence between the sub-genomes, an excess of heterozygosity is expected. Then, HomeoSplitter disentangles the two homeologous contigs based on the potential differential expression between the two homeologous copies of a given gene in the same accession. As the read counts may differ between homeologs according to their expression, the basic idea is that the most numerous nucleotides at all heterozygous sites of a given contig is likely to come from the most expressed homeolog. Taking into account the fact that the differential expression can vary among accessions is somehow more complex. HomeoSplitter tackles this problem by searching for the two contigs, obtained from the original one by modifying its problematic sites, which maximise the likelihood of the observed nucleotide frequencies under the assumption of differential expression between homeologs.

### Notations

**Figure 2:**
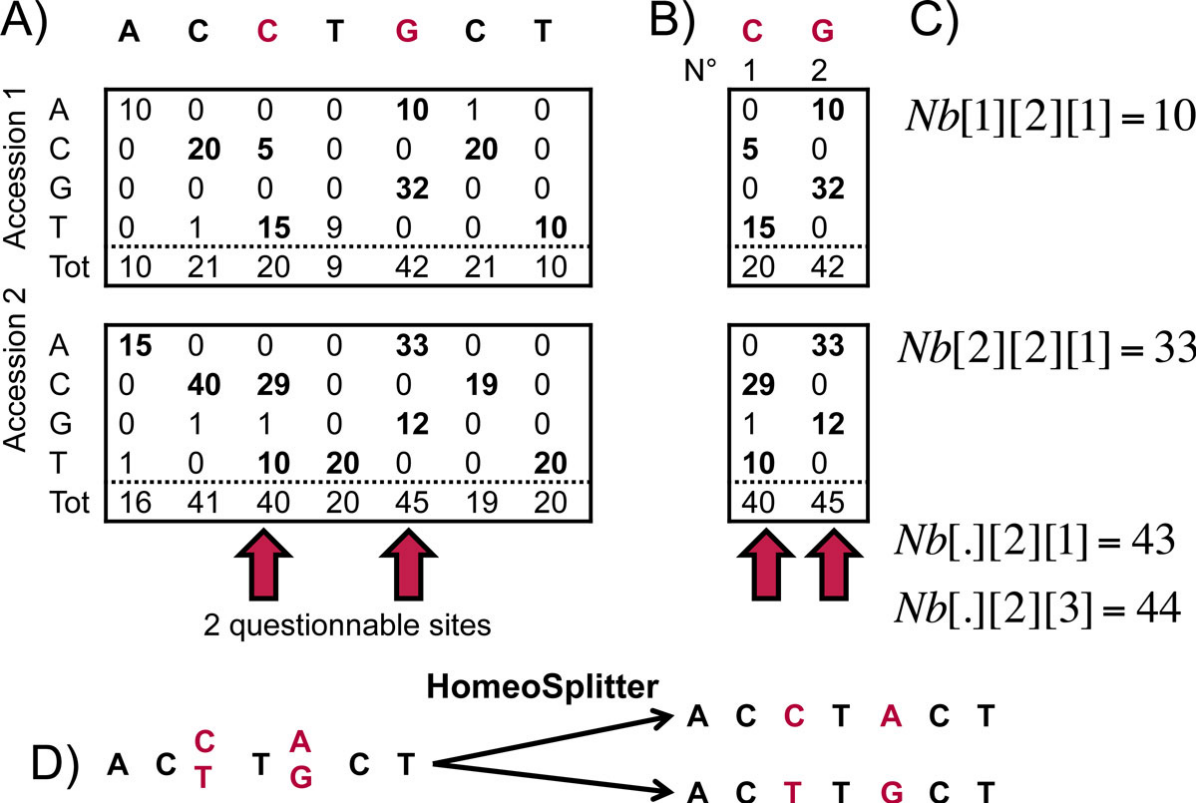
**Notations and key principles of HomeoSplitter**. Given a mapping on the contig (fragment) "ACCTGCT" one can count the nucleotides observed at each site for each accession (A). Questionable sites are those for which an excess of heterozygotes is observed (red arrows of A). Restricting the nucleotide counts to those questionable sites leads to the array *Nb *represented in (B). Even though homeologous copies are highly differentially expressed in each accession, considering them all at once here blur the signal. Indeed, at the second questionable site almost the same number of A (43) and G (44) are observed (C). To handle this problem, HomeoSplitter uses a specific expression bias for each accession; for instance considering the split defined by *C_k1 _*= [2,1] (*i.e*., pattern "CA") the estimated proportion of *C_k1 _*will be ~1/4 for the first accession (average of 5/20 and 10/42) and ~4/5 for the second one.

Let *Nb *be the array of *M*x*N*x4 cells containing nucleotide counts observed at *N *sites for *M *distinct accessions, such as *Nb[a][s][1]* (resp. *Nb[a][s][2]*, *Nb[a][s][3]* and *Nb[a][s][4]*) gives the observed number of nucleotide A (resp. C, G and T) of the *s^th ^*considered sites of *the a^th ^accession *(with 1≤s≤N and 1≤a≤M). Figure 2 provides example of such nucleotide counts.

Let *Mf1*_*Nb *(resp. *Mf2*_*Nb*) be the *M*x*N *array of the indices of the most frequent (resp. second most) nucleotides observed in *Nb *for each accession. For instance, if *Nb[2][1] = [0,29,1,10] as in *Figure 2B); then *Mf1*_*Nb[2][1] = 2 *(C is the most frequent nucleotide at the first site in the second accession) and *Mf2*_*Nb[2][1] = 4 *(*i.e*., *T*).

Note that counts from *Nb* can be considered globally (considering all accessions simultaneously) or locally for a given accession. To handle those different cases we will use the "." symbol to denote the fact that we sum over all possible value for this unspecified parameter (marginal sums). Using this convention then *Nb[.][s][.] *denotes the total number of nucleotides observed at site s for all accessions; *Nb[.][s][1]* denotes the total number of nucleotide A observed at site s for all accessions and so on (see Figure 2) C). Using analogous convention we denote by *Mf1_Nb[.][s] *(resp. *Mf2*_*Nb[.][s]*) the index of the most frequent nucleotide at site *s *over all accessions.

### Detecting questionable sites and contigs

We questioned the polymorphic sites obtained from the mapping of reads on the *de novo *contigs (see Figure 2). First, a site *s *is here considered to be heterozygous for one accession *a *if at least two different nucleotides *n_1 _*and *n_2 _*are each observed at least 5 times (*e.g*., sites pointed by red arrows in Figure 2A). More formally, those sites are those such as:

$$\exists n_{1,},n_{2}|1\leq n_{1}<n_{2}\leq 4;Nb\left[a\right]\left[s\right]\left[n_{1}\right]\geq 5;Nb\left[a\right]\left[s\right]\left[n_{2}\right]\geq 5$$

We then define a site as questionable if it is found heterozygous in at least one eighth of the genotyped individuals out of the 30 possible durum accessions. These two parameters can be tuned by users according to their biological datasets. The 5X coverage threshold limit the impact of sequencing errors and the 1/8 threshold is adapted to our almost fixed accessions. We consider the presence of questionable sites as an indication that this contig is indeed a mix of homeologous genes.

A contig is hence considered to be questionable if it contains at least one questionable site and has an average coverage of at least 5X ( $Nb\left[.\right]\left[.\right]\left[.\right]/N\geq 5$). Our approach assumes that each questionable contig mix reads from the two genomes of the studied tetraploid and could be hence split in two new contigs, identical for all sites but the questionable ones. Defining the split into two new contigs is thus equivalent to defining the two series of nucleotides observed at the questionable sites.

### Splitting questionable contigs

Having computed *Nb, Mf1*_*Nb *and *Mf2_Nb *arrays for the *N_q _*questionable sites of a contig *C_k_*, we can now try to disentangle problematic contigs by splitting them. A variant *C_k1 _*of *C_k_* is defined through an array of nucleotide of size *N_q_*, that defines the pattern used to replace nucleotide of *C_k _*at questionable sites (*e.g*., *C_k1_[2] = 1 *indicates that the second questionable site of *C_k _*will be replaced by an A in the new contig *C_k1_*). As we assume that questionable contigs are a mixture of two homeologous contigs, the complementary contig of *C_k1_*, denoted as *C_k2 _*is obtained by replacing the questionable nucleotides of *C_k _*by the most frequent nucleotides at this position once the nucleotide used in *C_k1_* has been excluded. More formally,

$$C_{k2}\left[s\right]=\left\{\begin{array}{c} Mf1\text{\_}Nb\left[.\right]\left[s\right];if\;Mf1\text{\_}Nb\left[.\right]\left[s\right]\neq C_{k1}\left[s\right]\\ Mf2\text{\_}Nb\left[.\right]\left[s\right];\text{otherwise} \end{array}\right)$$

A simple solution to split questionable contigs is to use the most frequent nucleotides observed at all questionable sites to build $C_{k1}$ (*i.e*., $C_{k1}\left[s\right]=\text{M}f1\text{\_}Nb\left[.\right]\left[s\right]$) and, hence, to use the second most frequent nucleotides to build $C_{k2}$ (note that by construction, this majority sequence corresponds to the *de novo *contig *C_k _*on which reads have been mapped). We call this first method the MajoritySplitter, this method assumes that the two homeologous genes are differentially expressed so that the most frequent nucleotides are likely to belong to the same homeologous sequence.

When considering several accessions, if the expression bias between the two copies is similar in all accessions, the signal is reinforced. On the other hand, if this bias varies from one accession to another, the final signal can be blurred when all individual data are merged altogether. For instance, if accession 1 has 1/3 of its reads from the genome A and 2/3 from the genome B when accession 2 has 2/3 of reads from genome A and 1/3 from B then on average, each genome provides 50% of the reads and there is no average differential expression. In such cases the majority sequences (and *de novo *assembled contig) can be chimeric contig mixing genome A and B (see Figure 2 for such an example). To ensure that individual signals add synergistically and not antagonistically, we computed the likelihood of the possible splits by explicitly using expression bias for every accession in each contig.

### Computing the likelihood of a split

The likelihood of the split defined by a pattern *C_k1 _*combines the likelihood of the pattern *C_k1 _*and the likelihood of its complement pattern *C_k2_*. The idea is that together they should provide a likely explanation of the nucleotide distribution observed at each questionable site. The ratio of the two homeologous gene copies in the sequenced cDNA is assumed to have influenced the ratio of *C_k1_, C_k2 _*reads that have been sequenced. The absolute number of reads sequenced at each questionable site may vary among sites but the ratio between those of *C_k1_, C_k2 _*is assumed to be roughly constant for a given accession all along the contig. We estimate the proportion of *C_k1 _*specific to an accession *a*, denoted as *p_1_[a]*, as the average of this proportion along all well covered sites:

$$p_{1}\left[a\right]=\underset{\begin{array}{c} 1\leq s\leq nbQuestionableSites\\ Nb\left[a\right]\left[s\right]\left[.\right]\geq 10 \end{array}}{\text{average}}\left(Nb\left[a\right]\left[s\right]\left[C_{k1}\left[s\right]\right]/Nb\left[a\right]\left[s\right]\left[.\right]\right)$$

The expected proportion of *C_k2, _*denoted as *p2[a]*, is then set as *p2[a] = 1-p1[a]*.

Given *p1[a] *and *p2[a] *the chance of having the observed nucleotide frequencies follows a binomial distribution so that the probability of site *s *of accession *a *given *C_k1 _*and *C_k2 _*under this model is:

$$\begin{array}{cc} p\left(Nb\left[a\right]\left[s\right]\text{|}C_{k1}\left[s\right],C_{k2}\left[s\right]\right)= & \left(\begin{array}{c} Nb\left[a\right]\left[s\right]\left[.\right]\\ Nb_{k1as} \end{array}\right)p_{1}{\left[a\right]}^{Nb_{k1as}}{\left(1-p_{1}\left[a\right]\right)}^{\left(Nb\left[a\right]\left[s\right]\left[.\right]-Nb_{k1as}\right)}\\ & \times \left(\begin{array}{c} Nb\left[a\right]\left[s\right]\left[.\right]\\ Nb_{k2as} \end{array}\right)p_{2}{\left[a\right]}^{Nb_{k2as}}{\left(1-p_{2}\left[a\right]\right)}^{\left(Nb\left[a\right]\left[s\right]\left[.\right]-Nb_{k2as}\right)} \end{array}$$

with $Nb_{k1as}\left(=Nb\left[a\right]\left[s\right]\left[C_{k1}\left[s\right]\right]\right)$ the number of occurrences at site *s *in accession *a *of the nucleotide proposed in the pattern *C_k1 _*at this site; $Nb_{k2as}$ the equivalent for *C_k2, _*and $Nb\left[a\right]\left[s\right]\left[.\right]$the total number of nucleotides observed at this site for this accession. Note that if $C_{k1}\left[s\right]$ is a nucleotide rarely observed at site *s*, corresponding to a sequencing error, the first term of the product may be quite high, but it will be counterbalanced by the second product term that will be quite low. Hence the necessity to consider both term simultaneously. For instance if there is a single accession and a single questionable site where 49 A, 50 C and 1 T are observed, the first term will be maximal when considering that $C_{k1}\left[1\right]$ is a *T*, since with *p_1_*= 1/100 it is highly probable to observe 1 T among 100 draws, but since it is also highly unlikely to observe only 50 C among 100 draws with *p_2_*= 0.99 this split will be discarded in favour to the much more likely explanation that $C_{k1}\left[1\right]$ is an *A *and $C_{k2}\left[1\right]$ is a *C*, or *vice versa*. Note also that these two equivalent solutions A/C or C/A do not have exactly the same probability under our model, and this will always be the case as soon as there are more than two nucleotides observed at the considered site.

Finally, we assume independence between sites so that the likelihood of $C_{k1},C_{k2}$ is just the product of site probabilities along sites and accessions:

$$Lk\left(C_{k1},C_{k2}\right)=P\left(Nb|C_{k1},C_{k2}\right)=\underset{s,a}{\prod }p\left(Nb\left[a\right]\left[s\right]\text{|}C_{k1}\left[s\right],C_{k2}\left[s\right]\right)$$

### Heuristic used to search for the maximum likelihood split

Our HomeoSplitter software offers two different strategies to determine the preferred split. The first one is an (almost) exhaustive search of the most likely split. For each questionable site we considered nucleotides present at least 5 times in at least one accession and test all combinations of those nucleotides. For each site the number of possibilities is thus greater or equal to 2 (otherwise the site will not be questionable) the total number of tested combinations is thus greater than $2^{N_{q}}$ for *N_q _*questionable sites. This solution is not tractable for large value of *N_q_*. Since the model assumes that the two homeologous copies are differentially expressed, it is reasonable to test the splits corresponding to the majority sequence of each accession (*i.e*., $C_{k1}\left[i\right]=Mf1\text{\_}Nb\left[a\right]\left[s\right]$). We also consider the split build from the most frequently observed nucleotides at all questionable sites (*i.e.*, the split returned by MajoritySplitter). As several accessions may have identical majority sequences, this leads us to design a heuristic search that tests at most (M+1) splits. As for $N_{q}\leq 10$ the full search was tractable, we compare both solutions on this subset of our benchmark. Since the same split is proposed by both methods in 99.6% we use the above described heuristic as the default strategy in HomeoSplitter without further attempt to better optimize our search strategy using a more complex strategy.

### Split similarity with the 2X reference sequences

To assess the performances of our HomeoSplitter method we compare the initial contig with the two resulting contigs supposed to represent the disentangled homeologous copies. This was done using the previously described benchmark, which focuses on *durum *contigs *C_k _*for which similar sequences have been found in each of the two diploid relatives, *urartu *and *speltoides*. The consensus sequence of the durum reads mapped on the diploid contigs (Figure 1) provides us with a "golden standard" of an ideal homeologous assembly hence, good splits are expected to have a good similarity with these two reference consensus. For the contig *C_k _*those two consensus sequences are respectively denoted as *S_k _*and *U_k_*. To assess whether splits obtained via HomeoSplitter get closer or not to this golden standard, we use the following criteria:

$$diffSym\left(C_{k}\right)=\text{max}\left(sim\left(C_{k1},U_{k}\right)+sim\left(C_{k2},S_{k}\right),sim\left(C_{k2},U_{k}\right)+sim\left(C_{k1},S_{k}\right)\right)-\left(sim\left(C_{k},U_{k}\right)+sim\left(C_{k},S_{k}\right)\right)$$

with *Sim *the similarity between two contigs defined as the percentage of identical nucleotides along their overlap. Note that the length of the considered overlapping stretches may be different with *urartu *and *speltoides *but they always spread over at least 100bp by construction of our benchmark.

As the max included in this formula may bias this measure toward positive values we also compared the performance of HomeoSplitter and MajoritySplitter to a random split of the contig (randomly changing the same number of sites, or randomly changing each questionable site with one of the observed nucleotides at this site).

### Genotyping, SNP detection and the *Fis *fixation index

The genotyping of each accession was done using reads2snp [35], which estimates the most likely genotype and its associated probability given a prior *Fis *value of 0.85. Under complete selfing, *Fis *should be set to 1 while under panmixia *Fis *should be set to 0. In our case, as durum lines were extracted from a mixed mating durum population, *Fis *was empirically set to 0.85, based on previous empirical estimates. We then conserve as reliable genotypes only those having, according to read2SNP, a probability greater than 0.99 and called on sites covered by more than ten reads.

At each polymorphic site (SNP), allele frequencies were computed on the set of properly genotyped individuals. From these values, *Fis *value was computed at each site. If *H_obs _*is the ratio of observed heterozygous individuals among the properly genotyped accessions for this site, then *Fis = 1 - Hobs/ (2 p (1-p)) *where *p* is one of the allelic frequencies (we kept only the bi-allelic SNPs here). *Fis *will be used as follows to validate SNPs and determine the efficiency of HomeoSplitter. In case of complete divergence between the two homeologs mixed in a single contig, all the genotypes will be heterozygous and *Fis *value will be negative (*Fis = -1 *since *Hobs = 1 *and *p = 1/2*). A good SNP in an inbred population such as durum wheat will have a *Fis *value close to 1 (*Fis = 1 *when no heterozygous genotype is observed). Intermediate situation may appear when one homeologous copy is monomorphic at one site (*e.g*., AA) when a derived allele is polymorphic on the other copy (*e.g*., AA or TT for inbred lines). In a mix contig, if *p *is the frequency of the T allele, ~*pM *accessions will appear as heterozygote AT (actually AATT) and (*1-p*) as homozygous AA (actually AAAA). In this homeologous/homologous polymorphic situation, *Fis = 0 *for *p = 1/2*. We thus expect *Fis *values to be distributed around three modes: close to 1 for valuable polymorphic homologous SNPs, close to 0 for a homeologous mix with a homologous polymorphism in one of the copies, close to *-1 *for SNPs revealing fixed divergence between copies.

A way to measure the efficiency of HomeoSplitter is thus to compare the distribution of the *Fis *of the *de novo *SNPs, HomeoSplitter SNPs and diploid SNPs.

## Results and discussion

### Software availability

HomeoSplitter is freely available at http://bioweb.supagro.inra.fr/homeoSplitter/ under the French CeCILL licence, which is compatible with the GNU GPL one. The software has been developed in Java and can thus be run on Windows, Linux and Mac Operating Systems. It takes as input an ALR file representing the frequency of each nucleotide in the initial mapping [35] and provides the new set of split contigs in fasta format as output. The SAM format is the *de facto *standard to represent mapping [36], the software SAM2ALR [35] allows to transform SAM formatted files into the ALR file that HomeoSplitter takes as input. All threshold values used to detect questionable sites and contigs are parameterized and HomeoSplitter also accepts a set of questionable sites as input. Starting from an ALR file containing reads counts for M accessions over a set of contigs, HomeoSplitter has a time complexity of *O(NM) *and a space complexity of *O(N_l_M)*; with *N_l _*the length of the longest contigs and *N *the sum of contig lengths. These low complexities allow to run HomeoSplitter on standard desktop computer; for instance our dataset of 3,709 contigs was processed in less than four minutes on a MacBook Pro laptop (OS X.6, 4 Go RAM, 2.3 GHz Intel Core i7 CPU).

### Considered dataset and benchmark assembly

The number of cleaned reads varies from 1.3 to 10.8 millions among the 30 durum accessions, with an average of 4.01 millions. *Speltoides *assembly was built with 20.25 millions cleaned reads and that of *urartu *with 26.7 millions. *Speltoides *assembly counts 16,891 contigs (sized by length > 500 bp) with an average size of 1,193 bp when *urartu *assembly has 21,907 sized contigs with an average size of 1,351 bp. The assembly of the durum reads coming from the 30 accessions yielded 27,820 *de novo *sized contigs of 1,055 bp on average.

After clustering (CAP3 90% of similarity), a total of 40,895 contigs (*urartu *+ *speltoides *+ *durum*) are present in 13,166 clusters. Durum contigs are present in 11,630 clusters (88%). The different clustering configurations are as follows: (durum + *speltoides *+ *urartu*): 6,057 clusters; (*durum*+ *speltoides *without *urartu*): 1,899 clusters; (durum + *urartu *without *speltoides*): 2,667 and durum alone: 1,007 clusters. Out of the 6,057 (*durum*+*speltoides*+*urartu*) clusters, only a subset of 2,505 clusters validated the conditions required for the benchmark of HomeoSplitter. These 2,505 clusters contain 3,709 durum wheat contigs, among which we kept only the 2,816 having an overlapping of at least 100 bp with simultaneously *urartu *and *speltoides *contigs.

### Improvement when disentangling contigs using HomeoSplitter

New contigs proposed by HomeoSplitter are closer to the diploid contigs than was the *de novo *contig in 2,506 out of 2,816 cases (89% of *diffSym(C_k_)>0*). In 6.2 % they were more distant (*diffSym(C_k_)<0*) and in 4.7% equally close (*diffSym(C_k_) = 0*). A paired t-test confirms that this difference is evidently highly significant (p-value<2.10^-16^). To control for the bias introduced by the max operator in the *diffSym *measures, we also consider splits obtained by choosing randomly at each questionable site one of the observed nucleotides. Both HomeoSplitter and MajoritySplitter produce significantly better values than this pseudo random splitting strategy (p-value of a paired t-test <2.10^-16^). Moreover, *diffSym *values obtained with HomeoSplitter are also significantly better than those obtained by the MajoritySplitter approach (454 cases better, 140 cases worse, *p-value *< 2 10^-16^). This result may be explained if the most expressed homeologous copy of one gene is not always the same among durum accessions, either because of genetic difference in gene regulation among accessions or from environmental differences during the growth and the sampling of seedlings.

**Figure 3:**
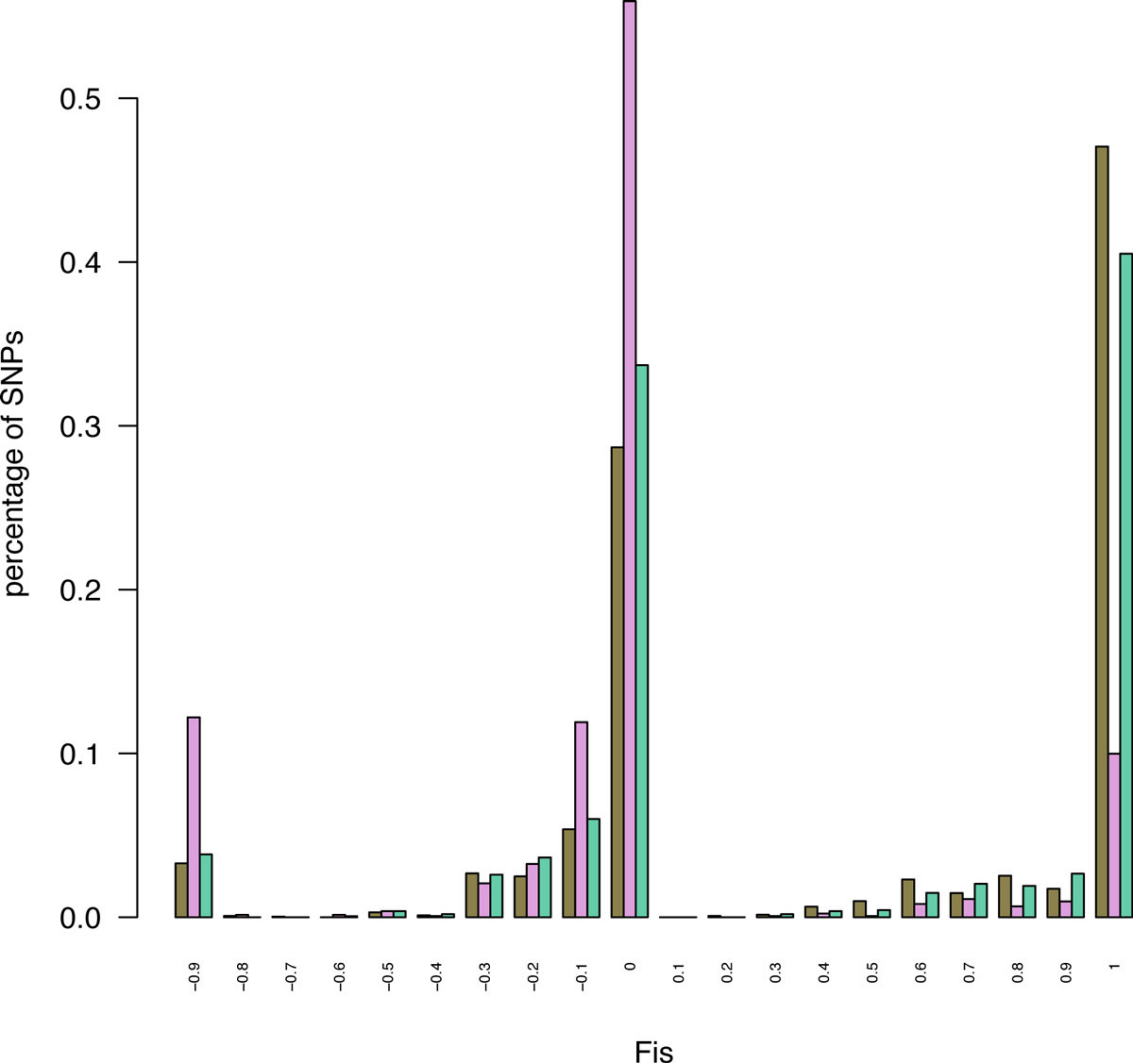
**Distribution of *Fis *values of: diploid SNPs (brown), *de novo *SNPs (pink) and HomeoSplitter SNPs (green)**. *Fis *values were calculated as the heterozygosity deficit relative to panmixia. Allelic frequencies were estimated from the called genotypes with high confidence values (see text). Negative *Fis *values suggest fixed divergence between homeologous/paralogous copies. *Fis *around 0 indicate possible mixture of homeology and intra genome polymorphism. *Fis *values close to 1 sign *a priori *intra genome polymorphic sites. See Figure 1 for the pipeline leading to the detection of these three SNP sets

**Figure 4:**
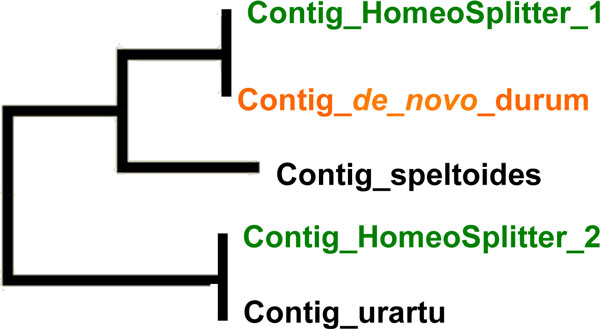
**Phylogenetic tree inferred on homologous contigs from cluster 6960**. The original contig obtained by *de novo *assembling (contig_*de_novo*_durum) was more similar to the speltoides contig (contig_*speltoides*) than to the *urartu *one (contig_*urartu*). After using HomeoSplitter we obtained the most expressed split contig (contig_HomoeoSplitter_1) that is still similar to the *speltoides *one and one less expressed contig (contig_HomoeoSplitter_2) highly similar to the *urartu *one.

The distributions of *Fis *obtained on the three mappings are plotted in Figure 3. *Fis *varies between -1 and 1 and the three expected modes are effectively observed. Mapping on diploid contigs and HomeoSplitter contigs produce very close results. Compared to the *de novo *case, using HomeoSplitter significantly reduces the proportion of negative *Fis *(*Fis*=-1 mode). This clearly indicates that HomeoSplitter separated correctly completely diverged sites between the two homeologous copies mixed in the original *de novo *durum contigs. The original contig is often highly similar to one diploid (the most expressed one) but reads from both copies are mapped on it. These cases are revealed by applying HomeoSplitter which splits it in two distinct contigs one being similar to *urartu *and the other to *speltoides*. This is illustrated in Figure 4 through the maximum likelihood phylogenetic tree of the different contigs present in the cluster number 6960. Conversely mapping on HomeoSplitter contigs greatly enhances the number of SNPs with high *Fis *values, particularly in the class above 0.9. The expected value of *Fis *of true homologous SNPs in our accessions is expected to be located around ca. 0.95. This proves that the excess of heterozygosity has been greatly decreased in this mapping compared to the mapping on the *de novo *durum contigs and that more "homologous" SNPs are now correctly detected. As *Fis *may vary, we empirically put a threshold value at *Fis *= 0.6 to accept a SNP as "homologous". We found 188 homologous SNPs out of the 1,360 SNPs detected in the mapping on the *de novo durum *contigs, i.e., only a relative fraction of 13.8 %. This ratio rocketed to 47% (762/1620) in the mapping on the HomeoSplitter contigs, reasonably close to the ratio obtained on mapping on the diploid contigs, i.e., 56% (1507/2661). A slight superiority of HomeoSplitter compared to MajoritySplitter is observed, the percentage of valuable homologous detection is only 45% in the latter case (710/1575) but this difference is not significant (*chi2 *= 1.23, *df *= 1, *p-value *= 0.73).

The discrepancy between the absolute numbers of SNPs detected in mapping on diploid (2,601) and on HomeoSplitter contigs (1,360) may be due to a difference of the considered fraction of the transcriptome. Indeed, on the 2,505 clusters of the benchmark, the summed length of contigs is ~4.85 Mb in diploids and only ~1.89 Mb in HomeoSplitter contigs (38.9%).

## Conclusion

HomeoSplitter relies on a straightforward algorithm based on nucleotide counts. This leads to an extremely efficient solution to deal with thousands of contigs in a matter of minutes. HomeoSplitter is efficient to rebuild two likely homeologous sequences from contigs on which mapping generates a large excess of heterozygous genotypes. Re-mapping reads on these new sequences leads to a huge gain of SNPs with *a priori *correct genotyping properties (*Fis *>0.6). In our benchmark, HomeoSplitter detects valuable SNPs for 47% of the detected polymorphism compared to the 13.8% initial mapping, the latter case being close to the ratio previously reported in the allo-tetraploid rapeseed [3]. This efficiency is only slightly inferior to the mapping on the diploid references (56%).

HomeoSplitter is expected to be efficient when a bias in the expression between homeologous copies exists in at least some accessions, whatever its distribution among accessions. Its power is strongly reduced in case of balanced expression. One planned extension is to warn the user in such cases that can be identified since several splits have almost identical likelihood. Furthermore, HomeoSplitter offers the possibility to seek for specific genome polymorphism to develop flanking primers for SNPs to be developed as markers on high throughput micro-arrays, hence possibly improving the genotype calling [8].

Computation time of HomeoSplitter is short and is thus a good complement of the approach relying on diploid reference transcriptomes [1,9]. Indeed, some genes may not be expressed or detected in one or the two diploid transcriptomes (or absent in the diploid genomes due to gene deletions subsequent to polyploidisation). In our case, since the reads number used for assembling transcriptomes was rather low, the benchmark including clusters of *speltoides, urartu *and durum contigs concerns only ~10% of the durum wheat contigs. HomeoSplitter appears as a convenient method for treating the remaining 90%. HomeoSplitter also avoids the fine tuning of stringency used for *de novo *assembly [1] and allows the use of a relatively permissive similarity for the assembly. This permits to keep all homologous alleles within the same contig, and to be able to examine the excess of heterozygosity *a posteriori*. As the divergence may vary a lot among genes (here the average similarity between *urartu *and *speltoides *is 97.5% with a standard deviation of 1.03%), a fine tuning of stringency is difficult to determine for a whole transcriptome (or genome).

Questionable sites were easily detected in the selfing durum wheat. For outbred species, the questionable sites may be detected by the likelihood approach used in the read2snp package [5]. It compares the likelihood of data for the existence of paralogs in diploid species with any kind of mating system. It may be further included in future version of HomeoSplitter. HomeoSplitter does not exploit the individual paired-end read information as in phasing methods [18-20]. A pipeline to disentangle homeologous copies based on such diploid phasing methods has just been published [37]. This method does not especially target questionable sites and does not exploit the well-documented phenomenon of expression level dominance in homeologous copies [26-28]. Mixing both approaches seems a promising solution; but HomeoSplitter offers already a rapid, simple and efficient solution.

## Competing interests

The authors declare that they have no competing interests.

## Authors' contributions

MTP and JD designed and managed the plant experiments. MA and SS prepared cDNA libraries from plant samples. GS and YH processed NGS data from raw reads to mapping. JD, VR, YH and SG conceived the homeoSplitter method, VR implemented it and YH conducted validation analysis. VR, MTP, JD, SG and SS wrote the paper, all authors read and approved the final version.

## Supplementary Material

Additional file 1 **This text file provides further details about the plant material**. Click here for file

Additional file 2 **This text file provides further details about RNA extraction and sequencing protocols**. Click here for file

Additional file 3 **This text file provides further details about contig assembly, read mapping and SNPs identifications**. Click here for file

## Declarations

The publication costs for this article were funded by the Agropolis Fondation under the ARCAD projet No 0900-001.

This article has been published as part of BMC Bioinformatics Volume 14 Supplement 15, 2013: Proceedings from the Eleventh Annual Research in Computational Molecular Biology (RECOMB) Satellite Workshop on Comparative Genomics. The full contents of the supplement are available online at http://www.biomedcentral.com/bmcbioinformatics/supplements/14/S15.
