# Supplementary material for: Disentangling homeologous contigs in allo-tetraploid assembly: application to durum wheat
Source: BMC Bioinformatics. 2013 Oct 15;14(Suppl 15):S15. doi: 10.1186/1471-2105-14-S15-S15 (PMC3851826; doi:10.1186/1471-2105-14-S15-S15)
Supplement: Additional file 1 — This text file provides further details about the plant material. [file 1471-2105-14-S15-S15-S1.PDF]

Plant material :

Durum wheat (*Triticum turgidum durum*) is the elite form of the *Triticum turgidum* tribe which derives from a wild ancestor *T. t. dicoccoides*. Genetic diversity in *T. durum* has been strongly reduced since domestication [37] [38, 39]). To create a new resource for durum wheat, we developed a base broadening population of durum wheat introgressing wild and primary wheat diversity into elite germplasm. To promote introgression and recombination, we artificially monitored the outcrossing at 10% using a male sterility gene since durum wheat is naturally inbreeding. The population has been founded in 1997 and is grown every year from a sample of the previous harvest of about 4000 seeds. For this study, 30 fertile mother plants were sampled in the population in 2009.
