# Supplementary material for: Disentangling homeologous contigs in allo-tetraploid assembly: application to durum wheat
Source: BMC Bioinformatics. 2013 Oct 15;14(Suppl 15):S15. doi: 10.1186/1471-2105-14-S15-S15 (PMC3851826; doi:10.1186/1471-2105-14-S15-S15)
Supplement: Additional file 2 — This text file provides further details about RNA extraction and sequencing protocols. [file 1471-2105-14-S15-S15-S2.PDF]

Data acquisition: from RNA extraction to sequencing

For durum wheat and *speltoides* samples, seedlings were grown from seeds in Petri boxes with 4 ml of purified water at constant temperature of 30°C in obscurity. Coleoptiles and primary leaves were sampled from 7 days seedlings, and crushed in liquid nitrogen liquid. For the *urartu* sample, mature plants gown in greenhouse were used to assemble a mix of 150 mg of young flower and leaves.

About 50 mg of tissue were used for each individual library. RNA was extracted using RNeasy Plant Mini Kit (Qiagen) with DNAse treatment, yielding from 30 to 60 µg of total RNAs. RNA quality was determined by the RIN (RNA integrity number) using the Agilent RNA 6000 Nano chip. RNA was quantified using the Quant-iT™ RiboGreen® RNA Assay Kit, and normalized leading to a RNA quantity of 2 µg per sample. Individual libraries were prepared using the TruSeq RNA sample Preparation v2 kit (Illumina Inc., CA), composed of different steps: selection, purification and fragmentation of mRNA (4 min to 94°C), reverse transcription, synthesis of DNA double strand, and ligation of individual adaptors included index sequences, in order to allow multiplexing. To increase sequences with ligated adaptors, enrichment was made by 15 cycles of PCR using PE1.0 and PE2.0 Illumina primers and with Phusion DNA polymerase (NEB, MA)

Each indexed cDNA library was verified and quantified using a DNA 100 Chip on a Bioanalyzer 2100 then equally mixed by 48. The final library was then quantified by real time PCR with the KAPA Library Quantification Kit for Illumina Sequencing Platforms (Kapa Biosystems Ltd, SA) adjusted to 10 nM in water and provided to the Montpellier Genomix platform for sequencing (http://www.mgx.cnrs.fr/).

Final pooled cDNA library was sequenced using the Illumina mRNA-Seq, paired-end indexed protocol on a HiSeq2000 sequencer, for 2 x 100 cycles. Each durum wheat genotype library represents 1/48 of one lane and *Aegilops speltoides* and *Triticum urartu* samples represent 1/12 of one lane of the flowcell.
