# Supplementary material for: Disentangling homeologous contigs in allo-tetraploid assembly: application to durum wheat
Source: BMC Bioinformatics. 2013 Oct 15;14(Suppl 15):S15. doi: 10.1186/1471-2105-14-S15-S15 (PMC3851826; doi:10.1186/1471-2105-14-S15-S15)
Supplement: Additional file 3 — This text file provides further details about contig assembly, read mapping and SNPs identifications. [file 1471-2105-14-S15-S15-S3.PDF]

Contig assembly, read mapping and SNPs identifications

Reads were cleaned using CutAdapt [40] (http://code.google.com/p/cutadapt/) to remove adaptor sequences, reads with mean quality score inferior to 30 and reads shorter than 35bp from raw data. *Urartu* and *speltoides* assemblies were realized for each durum line using Abyss with the *k-mer* parameter set to 60 [41] (www.bcgsc.ca/platform/bioinfo/software/abyss). Abyss contigs overlapping with a minimum of 60 bp were joined with the CAP3 DNA sequence assembler [42] with 99% of similarity.

D*e novo* assemblies of paired reads were done following the same Abyss Cap 3 procedure with 98% of similarity. Individual abyss Cap3 contigs shorter than 500 bp were discarded for further assembly but were kept for the mapping process to capture reads that may correspond to specific and annoying features such as sequences coming from alternative splicing, paralogs. A final Cap3 step assembled all individual durum transcriptomes in a single one with 98% similarity and 251 bp overlap (*i.e.,* greater than 500/2). Durum consensus, *urartu* and *speltoides* contigs were finally clustered using CAP3 (90% of similarity, overlap of 251 bp).

Mapping were done using BWA [43] (http://bio-bwa.sourceforge.net/) with a maximum of three differences per read mapped.
